# Social capital and wellbeing among Australian adults’ during the COVID-19 pandemic: a qualitative study

**DOI:** 10.1186/s12889-022-14896-x

**Published:** 2022-12-22

**Authors:** Heidi Green, Ritin Fernandez, Lorna Moxham, Catherine MacPhail

**Affiliations:** 1 Centre for Research in Nursing and Health, St George Hospital, Kogarah, NSW Australia; 2 Centre for Evidence Based Initiatives in Health Care: A JBI Centre of Excellence, University of Wollongong, Wollongong, NSW Australia; 3 School of Health and Society, University of Wollongong, Wollongong, NSW Australia; 4 Illawarra Health and Medical Research, University of Wollongong, Wollongong, NSW Australia; 5 School of Nursing, University of Wollongong, Wollongong, NSW Australia

**Keywords:** Social determinants of health, Social capital, COVID-19, Wellbeing

## Abstract

**Background:**

COVID-19 has created global disruption, with governments across the world taking rapid action to limit the spread of the virus. Physical distancing and lockdowns abruptly changed living conditions for many, posing specific challenges of social isolation and lack of connectedness due to being physically and socially isolated from family and friends. Social capital is the bonding of individuals within a society that facilitates and shapes social interactions. The aim of this study was to qualitatively explore the impact that existing social capital has on Australians’ experience of lockdowns during the COVID-19 pandemic and the effect this has had on their wellbeing and quality of life.

**Methods:**

Participants from various socioeconomic areas within Australia were purposively selected to participate in semi-structured interviews conducted via videoconferencing or telephone. Inductive thematic analysis of the data was undertaken.

**Results:**

A total of 20 participants were interviewed ranging in age from 21 to 65 years, including 50% (*n* = 10) females, 40% (*n* = 8) males, 5% (*n* = 1) non-binary and 5% (*n* = 1) transgender. Three main themes emerged from the analysis of the data: No person is an island; Social engagement; and Loneliness and isolation. Individuals who resided in low socioeconomic areas, those who lived alone and had reduced social support expressed feelings of poorer wellbeing.

**Conclusions:**

This study describes the lived-experiences of the influence of the COVID-19 pandemic on Australians’ social capital and wellbeing. The findings highlight the need for interventions to increase social support, social cohesion, and social connectedness, especially among Australians from low socioeconomic areas, to enhance their overall wellbeing.

## Background

Since emerging in December 2019 in Wuhan, China, SARS-CoV-2, otherwise known as COVID-19, has created global disruption, with governments across the world taking rapid action to limit the spread of the virus [1]. As part of the concentrated effort to curb the increasing number of people infected with COVID-19 and to decrease the number of severe infections, many countries imposed nationwide lockdowns [2, 3]. Massive scale lockdowns meant that travel was restricted, people were ordered to remain at home, quarantining for various regions, closure of businesses, schools and workplaces, reduction in public transport and work from home orders where possible [3, 4]. Physical (social) distancing and lockdowns abruptly changed living conditions for many, posing specific challenges of social isolation and lack of connectedness due to being physically isolated from family and friends.

In Australia, while much of the national focus has been on monitoring and controlling the spread of COVID-19, there is evidence in the literature that the effects of COVID-19 have been social and psychological [5]. The rise in COVID-19 infections, physical distancing regulations, lockdowns and disruption to daily life within Australia have heighten social isolation and loneliness impacting on the health and wellbeing of many Australians [5].

As a vital social determinant of health, social capital, defined as the conditions in which individuals *“are born, live, grow and work”* [6], provides a protective role in physical and mental health [6]. Social capital incorporates three relevant features: social support, social networks, and social cohesion. Social support is the direct help an individual receives through various social relationships. Social networks describe the people who are in an individual’s life and the relationships that exist between them, whereas social cohesion refers to the strength of the relationships either within a community or with friends and family groups [7]. In the literature, having good social support and social networks can safeguard against some of the negative effects of other social determinants of health such as poverty [8], and can lessen the vulnerability of people who are located lower on the social gradient [9]. Despite this potential, individuals with diminished economic capacity are sometimes unable to avail themselves of certain social capital or are excluded from social networks or participation and can therefore experience a negative effect on their health [10]. Social capital plays a key role in shaping social and economic outcomes, and research has demonstrated that societies with higher social capital have higher incomes, are less corrupt, are healthier, and function better [11]. In fact, there is a direct association between social capital and health, with strong social capital correlated with health information sharing among family members and higher self-rated overall health [12, 13]. Indeed, social capital has the ability to improve economic efficiency through coordination and cooperation of shared norms to grow entrepreneurial firms, engage in technological advances and enhance strategic alliances [11]. However, it is imperative to note that varying levels of social capital can produce unequal impacts on social and health outcomes, as it means differing resources and support [14].

Within the context of the pandemic, those who were socially disadvantaged or had low social capital prior to the pandemic are more likely to have experienced detrimental effects on their health and wellbeing. There is a direct association between social position and stress, with stress a result of coping with other social determinants such as poverty, housing instability, unemployment, and intergenerational disadvantage [15, 16]. Additionally, social distancing and lockdown measures in response to the pandemic have limited social interaction, with previous epidemics demonstrating rises in loneliness and psychological consequences such as anxiety and depression [3]. Furthermore, the impact of lockdowns have seen an alarming increase in domestic violence incidents globally due to social isolation [17], affecting wellbeing and mental health and driven by those residing in a low socioeconomic areas, and among those with financial difficulties [18].

Despite there being a wealth of quantitative literature exploring the impacts of the social determinants of health, such as social capital, there is limited post positivist evidence - recognising subjectivity and avoiding bias - examining the lived experiences of individuals. Therefore, this study aims to qualitatively explore the impacts that existing social capital had on the experiences of Australians in lockdowns during the COVID-19 pandemic and the effect this has had on their wellbeing and quality of life.

## Methods

### Design

This descriptive qualitative study is underpinned by Sandelowski’s [19] classification of qualitative descriptive design methods, which from a philosophical perspective draws upon naturalistic inquiry and interpretative study designs. A qualitative descriptive approach provides an opportunity to explore and gather a broad insight into the phenomena of interest, which is particularly indicated when little is known on the topic [20]. This is pertinent in a study that aims to explore how existing social capital impacts the experiences of Australians during lockdowns during the COVID-19 pandemic and the effect this had on their wellbeing. This approach enables a rich understanding of the participants’ experiences and perceptions. This study is embedded within a nationwide mixed methods study investigating the relationship between wellbeing and social determinants of health among Australians during the COVID-19 pandemic.

### Participants and recruitment

Purposive sampling was used to ensure a comprehensive cross section of participants and representativeness of remoteness, socioeconomic status, gender, age and state and territory of Australia. Participants who had completed an online survey as part of the larger mixed methods study and agreed to participate in the qualitative component of the study were eligible for purposive sampling. A detailed description of the recruitment process for the online survey is reported in [21]. To achieve remoteness sampling for the online survey, the primary researcher (HG) used the Australian Bureau of Statistics (ABS) remoteness structure, Accessibility and Remoteness Index of Australia (ARIA+), that enables the user to target major cities, regional and remote locations. Socioeconomic sampling for the online survey was achieved by using the ABS Socio-Economic Indexes for Areas (SEIFA) Index of Relative Socio-Economic Advantage and Disadvantage (IRSAD) maps, which enable the primary researcher to use postcodes to select participants based on their socioeconomic status. The IRSAD is used to collate data on individuals’ social and economic conditions by local government area, providing a score of either advantage or disadvantage. A high score indicates greater socioeconomic advantage, and a low score specifies greater socioeconomic disadvantage [22]. This score has been used to classify participants in the study as either from a high or low socioeconomic area.

All participants that agreed to be contacted for the qualitative component of the study provided their contact details in the online survey, confidentiality of these participants was achieved by providing them a study code. Once assigned a study code, all contact details were removed and kept in a password protected file by an independent researcher. Using the sampling framework, potential participants were purposively selected by the primary researcher. Potential participants’ study codes were then provided to the independent researcher who gave the contact details of the corresponding study codes to the primary researcher. Potential participants were approached through their email addresses and were provided with information regarding the study and a consent form to return should they agree to participate.

### Data collection

Semi-structured interviews were deemed the most appropriate method of data collection to meet the study aim and to provide a broad insight into the relationship between wellbeing and social determinants of health among Australians during the COVID-19 pandemic [23]. Informed by the results of the quantitative analysis is [24] and extensive review of the literature [25], a semi-structured interview guide was designed to investigate the ‘why’. The semi-structured interview guide contained open-ended questions such as ‘Please tell me about your experiences during COVID-19?’, and ‘Please tell me about any circumstances in your life that you feel impacted your experience of COVID-19’. Prompting questions were also used to generate further discussion and explanation from the participants, when required.

Due to the geographical dispersion of the participants, the one-on-one semi-structured interviews were held either by videoconference or telephone. Despite the primary researcher’s preference for conducting the interviews via videoconference, some interviews were held on the telephone due to slow internet bandwidth or no camera options available to the participants. Telephone interviews were held with three participants. All interviews were conducted at a mutually agreed time and date between 12 March 2021–28 August 2021. The semi-structured interviews were conducted by a female PhD candidate and the primary researcher on the study (HG) who is a public health professional with previous experience in descriptive qualitative interviewing. Before conducting the interviews, the study details were emailed to the participants, with all participants understanding that their participation was voluntary, and they had the option of withdrawing from the study at any time. A signed consent form was returned to the primary researcher prior to the commencement of the interviews. All semi-structured interviews were audio-recorded, with field notes taken during and following each interview. Each of the interviews with the participants ranged from 30 to 60 minutes. A $50 grocery gift card was provided as a gratuity to each participant in recognition of their time. The funds for the monetary incentive were from the primary researcher’s personal funds. Semi-structured interviews continued until data saturation had been achieved [26].

### Data analysis

An inductive thematic analysis as described by Braun and Clarke [ 23] was used to analyse the data. Instead of the researcher assigning their predetermined ideas, the inductive thematic approach allows for meaning to be originated from the content of the data. To ensure anonymity, each participant was provided with a pseudonym and the semi-structured interview audio-recordings were then transcribed verbatim by a professional transcription service. Once transcribed, all audio-recordings were re-listened to and checked against the transcripts to ensure accuracy. To assist with data analysis, all transcripts were imported into NVivo 12. Using the inductive thematic analysis approach, the first step was immersion within the data, reading and re-reading the transcripts and listening to the audio recordings. Secondly, initial codes, meanings and patterns were generated. As the analysis progressed the initial codes were arranged into potential themes, with coded extracts collated. To ensure the potential themes remained grounded in the data [27] and resembled the data, the coding framework was reviewed and checked against the transcripts. From the themes, sub-themes were identified that described and summarised the data. Each theme and sub-theme were refined to ensure it reflected the patterns and meanings within the entire dataset.

### Ethical considerations

Ethics approval was received from the University of Wollongong  Human Research Ethics Committee (HREC) approval no: 2020/306, prior to commencing this study.

### Rigour

To ensure rigour, the criteria of trustworthiness and quality as explained by Lincoln and Guba [28] were used. Checking the accuracy of the data and ensuring data saturation had occurred established the credibility. A diverse sample of participants from various socioeconomic areas that were geographically dispersed enabled transferability. Dependability was established by the research team engaging in frequent open discussions about the interpretation of the data. Establishing ongoing reflexivity throughout the research process allowed for confirmability to be achieved.

## Results

**Fig. 1:**
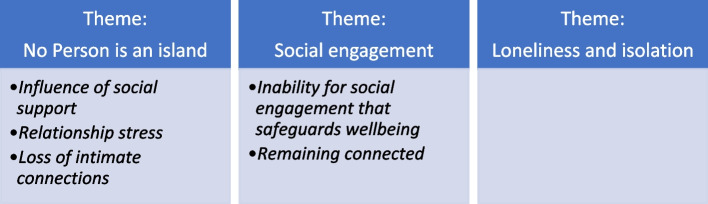
Themes and Subthemes

Twenty participants were interviewed from a range of socioeconomic areas across Australia. Participants varied in ages from 21 to 65 years, with 50% (*n* = 10) identifying as females, 40% (*n* = 8) males, 5% (*n* = 1) non-binary and 5% (*n* = 1) identifying as transgender. Participants were geographically dispersed across all states and territories and from a variety of socioeconomic areas within Australia. Data analysis revealed three themes: No person is an island; Social engagement; and Loneliness and isolation. Verbatim quotes from the participants in this study have been used to illustrate the key themes. Quotes used in this study were chosen based on the best representation of the experiences that matched the main themes. Reported quotations are followed by a pseudonym to guarantee participants anonymity. The themes are discussed in detail below and in Fig. 1.

### No person is an island

Concerns regarding social connection were voiced by the majority of participants in this study, with lockdowns creating a social void in their lives, a desire among some for human touch, relationship stress among some couples, while others felt a lack of social support during the pandemic. Physical distance between friends and family was often expressed as ‘anxiety inducing’ and a challenge.

#### Influence of social support

There were clear differences in the experience of social support based on living arrangements and socioeconomic status at the start of the pandemic. Some participants expressed that they had received adequate social support, while others felt that their social support was distanced or taken from them, and others lacked social support altogether. Living in a share house provided some participants with a familial social support, with one participant, “Sergio” expressing:

“I was really, really lucky to have a supportive familial relationship in my share house. So we really looked after each other. So there was that solidarity by all of us sharing together and we have each other and we would find ways to entertain ourselves.” (Sergio, aged 35).

Despite this type of social support considered positive, for some it did not replace the social support received from friends or others, saying “I mean, I have been described as a social butterfly and an extrovert so there was support but there wasn’t enough and that’s me, so yeah”. (Sergio, aged 35). In contrast, being an international student who recently arrived in Australia while living in a share house that had no social interaction was difficult and isolating, with one female participant, “Haimi” explaining that:

“They [house mates] were very stressed and we’d hardly talk to each other. No one wanted to have a chat, so I think that’s when I felt really isolated, because I was in that house all the time and I didn’t have anyone to talk to” (Haimi, aged 25).

It was a particularly difficult time for those who were isolated from family and friends, feeling their social support was removed from them saying “It meant that I couldn’t see people face to face, and because so many of my friends are interstate, it did mean that I was cut off largely from them.” (Reuben, aged 61). Living in a rural area, with a lack of access to social support while having to endure a miscarriage was particularly challenging and distressing for one female participant, “Clara” who said:

“With friends, that basically just evaporated. Everybody was locked down and stressed and really, I lost touch with just about everybody. I mean, other than my mum, I had no one. That was pretty tough, to be honest, because in a lot of ways, when you’re in rural areas, you rely on your social supports rather than anything else and that just wasn’t there. I mean, it was but it’s just kind of not the conversations that you can really have over Facebook, you know?” (Clara, aged 38).

While “Clara” had support from her mum, she felt awkward discussing her grief saying,

“I felt really awkward because she [mum] spends all day dealing with people who’ve got significantly worse problems so I didn’t want to add to that. So I mean, when she [mum] gets home, she doesn’t need to continue working. So if I was having a bad day or something, I just kept it to myself.”(Clara, aged 38).

However, other participants expressed receiving adequate social support and discovering who ‘true’ friends were. One participant, “Manaia” said:

“I really found who my friends are. Some of them, and it was much to my annoyance at the time, but some of them just made a real effort to make sure that they knew I was okay and then others I now see as fair-weather friends, if you’ve heard of that term. The ones I thought were my true friends I know are my true friends and they were really there for me and did what they could to help.” (Manaia, aged 52).

For those that lived with their partners, they expressed sufficient social support being able to connect with each other but also maintain a connection with friends. One participant, “Trey” elaborated on this saying:

“We are both quite capable of becoming homebodies if need be. We have the dogs, we’re quite content with each other’s company a lot of the time, play computer games, boardgames, talk. I wouldn’t say our friendships suffered at all. We kept in contact with each other. I made a point of making phone calls, which we almost never do. We don’t use telephones. But I made a point of actually ringing my friends, at least once a month just to check how they’re going, make sure things are fine.” (Trey, aged 40).

This was a similar experience expressed by another participant who said “I think my social support is pretty strong, so that’s pretty good. I’ve got friends, family, husband and then now I’ve got some workmates as well in the hospital because we - so that’s a lot of support as well.” (Xiuying, aged 21). Furthermore, others felt that social support was available to them if they required it, with one participant saying, “I don’t think I’ve had any particularly lack of support in any one direction so I suspect if there were people I needed to talk to I could.” (Marcel, aged 51). Participants who resided in high socioeconomic areas and in geographic locations in which strict lockdowns were not imposed, did not experience the lack of social support that other participants felt, saying, “Well Canberra didn’t really - we didn’t go through any kind of lockdown, really. So we haven’t had that experience. So really, those patterns of - those social patterns and social support didn’t change too much from our regular activities” (Parrie, aged 64).

#### Relationship stress

Although some participants felt they had adequate social support, others expressed relationship stress due to changes in their living arrangements, other stressors and anxiety. This was expressed among participants, regardless of their socioeconomic status. One female international student, “Mandeepa” expressed her concerns regarding her relationship with her partner saying:

“Because we had never lived together or had that and putting two people that are in a long-distance relationship in one confined space does not really go well. Definitely it took a huge toll on our relationship. I was at the point where I’m like, okay. My thesis is dependent on him. Things are just not going okay. I’m going to have to go back home. Yeah, I was prepared to go back and figure out a new life, and everything.” (Mandeepa, aged 26).

While for other participants the anguish caused by border closures and fear of spread of COVID-19 meant that they experienced relationship tensions because of extreme concerns and anxiety. One female participant who had family overseas explains “Like, around April, May, June, it was quite - I don’t know the word, but like my partner and I had a lot of relationship challenges as a result of me just being super-irritable and panicky and anxious.” (Emma, aged 31). Furthermore, lockdowns and stay at home orders forced couples to be confined to their residence precipitating relationship stress, with couples arguing. One non-binary participant, “Trey” said:

“I function from day-to-day quite fine, there’s no domestic violence. I say I’m arguing with my partner, but this is for the first time in 10 years of a relationship. We’re not serious arguing, we’re not fighting. We always make up by the end of it. Although, I think we’re both aware that it’s something we need to deal with, it’s not like we’re looking at the world collapsing down around us.” (Trey, aged 40).

#### Loss of intimate connections

A lack of intimate human connection during the COVID-19 pandemic was a common experience felt among the participants in our sample, which was most prominent among those who resided in low socioeconomic areas and didn’t live with a partner or were occupants of a share house. One international student who lived in a share house expressed how she missed human physical touch saying:

“like sometimes you just really crave physical touch. I just wanted someone to give me a hug. I could talk to them, but I just really wanted a hug, or I really wanted to just sit with someone and play boardgames, or just do something together.”(Haimi, aged 25).

While for another participant who had recently lost her partner to an unexpected death and had also lost her employment due to the pandemic, she felt she needed the human connection and comfort of her friends stating “That was when I was really needing my friends. Not having a lot to do and having to try and find things to keep me occupied rather than getting into my own head.” (Manaia, aged 52). The loss of human connection was associated with poorer wellbeing for many participants, particularly those who resided in low socioeconomic areas and among international students. One international female student participant explained her psychological wellbeing after being geographically separated from her boyfriend:

“Oh, it’s really hard. As I told you, when I went here, to Victoria, I felt like I have separation anxiety. Because I was crying every day, every night. Everything little thing I’ll remember about him when we’re together. We just sometimes really want to be with each other, human touch and talk about things, which we cannot do. We’re just on Zoom call. It’s hard.*”* (Kailani, aged 26).

This was a similar experience for “Nick”, who was in a long-distance relationship. Being geographically distanced from his partner affected his human connection during the pandemic, he expressed his concerns as.

“My partner actually lives interstate, it’s a bit of a long-distance relationship. The travel bans affected that interaction and connection. Missing out on going on holidays. We’d planned to go overseas and all that kind of stuff. It’s also delayed our plans about marriage and living together as well.” (Nick, aged 52).

For other participants, the lack of ability to leave the house beyond the restricted 5 km radius, was challenging especially for those who were single and used social events to meet potential partners. The lack of social events led to non-existent intimate human connections for some, with one participant expressing:

“I feel like that led to a lot of yeah, just a lack of human touch. A lack of actual engagement with my fellow human beings as we share this space. So that made it really difficult and socialising and having a social outlet and even meeting people was just unimaginable”. (Sergio, aged 35).

Others used animals as a substitute for human connection, with Manaia elaborating “One of my friends’ dogs had puppies, so I ended up with one of the puppies. That gave me the companionship I was really missing. She gave me the puppy as a foster situation but I think she knew that she was never going to get it back.” (Manaia, aged 52). However, for Parrie who did not live with his partner, he felt cautious when it came to sexual intimacy due to the concerns around the spread of COVID-19, with him saying “Of course, health and safety is always a priority in that regard but I suppose intimacy has been an issue as well. With my partner. Although that sort of has relaxed a bit. Initially, we were very wary about all that.” (Parrie, aged 64).

### Social engagement

Stay at home orders limited social interaction and social engagement among participants in this study. Social events and outlets were almost non-existent for many during the height of the lockdown period and in the time following, due to fear of spread of COVID-19. The lack of social engagement affected many participants’ wellbeing and quality of life, this was especially noticed by those who resided in low socioeconomic areas, lived alone and were from regional areas.

#### Inability for social engagement that safeguards wellbeing

The absence of any social engagement was described by participants as affecting their wellbeing, leaving them feeling lonely and desiring social interaction. For one transgender participant who lived alone in regional Australia, the community event that she joined on a weekly basis was cancelled, she expressed the impact this had on her wellbeing saying:

“It wasn’t too good. I mean especially because most of the time I am alone at home, so that as pretty much the only outing that I’d have during the week, apart from just going shopping. But yeah, it was a bit lonely.” (Nyah, aged 46).

For “Reuben”, who also lived alone and engaged socially through interstate travel, the closure of borders meant that he was unable to socially interact. This had a significant impact on his wellbeing as he was already experiencing mental health issues, with him saying “Because I wasn’t getting to travel, there was nothing that would give that bit of a bump in my motivation or my mood, so there was nothing that would break that cycle, so it [wellbeing] was worse from that point of view.” (Reuben, aged 61). Furthermore, lack of social engagement for participants that lived alone in a low socioeconomic area influenced their ability to cope, with a male participant stating:

“I don’t have a huge amount of friends but just the social interactions that you miss. I do a weekly catch up with a group of mates. I’d go over to a mates place to watch some footy or car racing and stuff like that. That was all cancelled. That was the sort of impact but just the lack of social interaction I guess” (Nick, aged 52).

The lack of a social outlet was challenging for Manaia, whose partner had recently passed away, while she needed to grieve, not being able to engage with others left her lonely with her mental health declining. “Manaia” said “Yeah, and that’s why I was quite lonely, because we had periods of time where we weren’t allowed visitors.” (Manaia, aged 52).

#### Remaining connected

While the ability to engage in physical and face to face social interaction was limited, many participants in this study found alternative methods to remain connected with family and friends, which assisted in their overall wellbeing. Many participants described ‘catching-up’ with family using videoconferencing services such as zoom and FaceTime to remain connected and replicate some sort of normalcy. Remaining connected through technologies was an experience often expressed by participants who resided in high socioeconomic areas. Using zoom was a common alternative used by families with one male participant saying, “I remember at the beginning the lockdown in Melbourne I had a weekly Zoom catch up with the whole family” (Marcel, aged 51). While another male participant, “Joshua,” explains:

“During the actual lockdown, we set up video calls, we had group family calls, we were all chatting away and we’d just have in the background and the kids would play at each other, in a sense. We set up video calls for the kids with their cousins so that they would have a phone or an iPad with Facetime and be playing in their room with one of their cousins doing the same thing in their house” (Joshua, aged 43).

“Alicia’s” family had never used zoom to hold family meals together, however adopted this approach to stay connected during the lockdown, saying:

“We’ve never done a Zoom meeting or anything like that, so for that benefit it was nice. We were doing it weekly with the whole family and it was - we made it a bit of fun. We all did our favourite dishes and it was nice. I think, if anything, we probably communicated more rather than less” (Alicia, aged 31).

Social media was also a popular medium used to keep connected with friends, with one participant saying *“*Then friends as well, because of the restrictions I used to see them once a week as well, so now I haven’t seen them for months. Definitely do really miss them but we just keep in touch via social media.” (Xiuying, aged 21).

The border restrictions on overseas travel meant that being at the birth of her first grandchild was impossible for “Manaia”, however, she explains that Skype was used to enable her to still experience the birth in real time saying:

“No, I haven’t met my grandchild yet. I would have liked to have been there for her labour too and when she came home with bubby. But my mother-in-law - her mother-in-law has been fantastic and they sent me lots of videos and I was on Skype with them while she was in labour. So I was as close to being there without being able to be there. I was very grateful for technology. It just made it a lot easier. But it will be nice when they come over and see me.” (Manaia, aged 52).

Technology and digital interaction were seen as tools for which participants were still able to engage and interact with friends and family, with one participant stating “Yeah Messenger and Skype and WhatsApp were at the top of your priorities list. We’ve now got new family groups on Messenger.” (Aaron, aged 65).

### Loneliness and isolation

Loneliness throughout the lockdown periods was experienced as an outcome of lack of in-person social interaction. Participants felt that loneliness was due to the isolation they experienced due to physical distancing measures and lack of human connection, which was often experienced by those who reported being affected by other social determinants of health. Living alone was consistently raised by participants as a contributing factor to their loneliness, however this was often associated with the exacerbation of other social determinants of health, including loss of employment and loss of income. One female participant elaborates saying.

“I found Covid quite lonely. I had gone from living with my partner to being alone. I was dealing with grief and I found that going to work was really good for me. Then when there was no work not only was I dealing with grief, I was dealing with the fact that I wasn’t entitled to any benefits at the time because I’m not an Australian citizen and New Zealand - my visa makes me ineligible for Centrelink [government] support. So things were quite stressful and dealing with grief on top of it and not being able to see my friends”. (Manaia, aged 52).

Loneliness and isolation were exacerbated for some participants during the pandemic, with “Reuben” stating “Yeah, I felt pretty isolated and lonely. But then, as I say, I feel isolated a lot of the time, but it gets broken up normally.” (Reuben, aged 61). Feeling socially isolated and lonely was mentioned by one female international student participant as stemming from the lack of social cohesion within the share house, saying “I think there were feelings of isolation and loneliness there too, because of the house situation, because I didn’t get the social life as much at that point.” (Haimi, aged 25). The isolation from social networks and social support, as well as the loneliness caused one participant to resort to taking drugs as a way of coping, saying “to be honest, I may have broken out some of the prescription drugs that were around the house every so often.” (Clara, aged 38). Similarly, the stress and isolation from social networks intensified others addiction behaviours, leading to poorer wellbeing, with “Trey” saying.

“I’m drinking probably the better part of a bottle of vodka a day now. That’s not entirely lockdown, but definitely coronavirus and some of the stresses associated with that have exacerbated my drinking, I believe. It is definitely part of the way of coping with social isolation and what’s happening*.”* (Trey, aged 40).

## Discussion

Individuals’ behaviours and social relationships are embedded within communities and neighbourhoods, therefore social capital provides a valuable perspective on the understanding of how social environments can influence health outcomes. This study provides new evidence for understanding the influence that multiple components of social capital have on the wellbeing of the Australian population during the COVID-19 pandemic. Three themes emerged from this study, no person is an island, social engagement and loneliness and isolation.

Perceived or actual access to social support provides a protective factor against negative life events, both in terms of psychological and physical health, enabling individuals to feel in control of stressful life situations [29, 30]. While social support varied among participants in this study, most expressed concerns regarding inadequate social support during the pandemic. Those who lived in low socioeconomic areas, those who identified as female and among international students were particularly likely to note this. According to social scientist Putnam, in those communities that have high social capital individuals do things together, such as church, membership of organisations and simply, doing activities together such as bowling [31].A study by Borgonovi and Andrieu found that communities that were able to join together to do social activities *(“bowl collectively”*) prior to the pandemic, those with high social capital, were able to do activities alone *(“bowl alone”)* to a greater extent during the COVID-19 pandemic [32]. Similarly, this study found that those with higher levels of social support were buffered from difficulty in coping and poor psychological wellbeing, compared to those with poor levels of social support. Literature examining the mental health of individuals during the COVID-19 pandemic found that high social support was a protective factor against stress relating to crises, with being female and worsening finances predictors of stress [33].

Additionally, this study found that those who resided in low socioeconomic areas expressed poor social support compared with those living in high socioeconomic areas and that residency in low socioeconomic areas was also associated with a loss of human connection during the pandemic. Furthermore, women and international students also conveyed poor social support, which could be a reflection of their social support prior to the pandemic as well as an exacerbation of their existing social determinants of health including ethnicity, employment, poverty and income. This is unexpected given that unemployment and housing insecurity are factors associated with a lower socioeconomic status [34], however maybe a reflection of the type of participants recruited into the study.

Social engagement, immersion in community and a sense of belonging are vital for human wellbeing and health [35, 36]. Similar to Putnam’s explanation of social capital, communities that demonstrate solid social connections, relationships and engagement, also benefit from greater individual wellbeing [31]. In this study, the lack of social engagement and social connections significantly impacted the wellbeing of individuals residing in low socioeconomic areas, those living alone and from regional areas within Australia. Previous studies have shown that socioeconomic status affects patterns of social capital and that individuals with higher incomes, education and occupational status are more often involved in volunteering, belong to political parties and other organisational groups, and therefore have higher social capital [37–39]. This often reflects social inequalities that place constraints on the ability of and opportunities for individuals from lower socioeconomic areas to immerse themselves within the community.

Having strong social capital fosters a sense of belonging and provides meaning to life, therefore enhancing an individual’s overall wellbeing [40]. This study has found that individuals within Australia who resided in high socioeconomic areas were still able to remain socially connected while enduring the isolation of lockdowns. Remaining connected through technology was vital for their wellbeing and ensured some sense of normalcy during the pandemic. Participants in this study used alternative methods to remain connected with their family, friends and community providing them with the necessary support required to assist them through the difficulties of lockdown. This echoes the findings of a study conducted in the United States among older people at risk of isolation and loneliness, which demonstrated that adoption of technology, including video calls, significantly reduced loneliness measures and significantly increased emotional wellbeing [41]. It is clear from this study that having strong social support and networks prior to the pandemic enabled individuals to adapt to ensure their psychological wellbeing was maintained. However, access to and ability to pay for technologies to stay connected was also an important factor and may have been restricted by socioeconomic status.

Lack of social interaction exacerbated loneliness and isolation among those from low socioeconomic areas and those who lived alone prior to the pandemic. Indeed, a disadvantaged social status has only amplified the effects of the pandemic. To cope with the social isolation and loneliness of the pandemic some participants in this study resorted to using drugs and alcohol, further decreasing their mental wellbeing. This finding is consistent with US findings in the literature, which have shown that there is a direct relationship between loneliness and alcohol consumption, with the COVID-19 pandemic increasing solitary alcohol consumption [42]. The same study also noted that social support is a protective factor for excessive alcohol consumption [42]. Similarly, research from the US has shown that increased drug use during the pandemic was associated with elevated levels of loneliness and anxiety [43]. This study has provided evidence demonstrating the mental health and wellbeing consequences that a lack of social capital and social support has had on vulnerable individuals during the pandemic. The results of this study add to the body of evidence regarding the increase in loneliness within the twenty-first century [44–48], not just during the pandemic. However, evidence-based interventions to address loneliness are limited. Social prescribing is one intervention that has been used throughout the UK to address loneliness, and this model connects an individual with a support worker for a short time period, to assist them in connecting with community groups and activities. While not primarily used for loneliness, some limited studies have demonstrated that social prescribing is successful in addressing loneliness [49, 50]. The findings of this study demonstrate the need for social isolation and loneliness to be address through interventions such as social prescribing. It calls for renewed action on the social determinants of health for the immediate and long-term future. Evidenced based interventions to address social support, social cohesion and loneliness are urgently required.

### Limitations

The scope of this study indicates a potential for responder bias towards individuals with an interest in COVID-19, despite participants being purposively selected. We took steps to ensure a diverse sample in terms of age, gender, socioeconomic status, and geographical location to ensure a wide range of Australian adults’ experiences were received. Given the qualitative nature of this research, is the results are not intended to be generalisable, but instead seek to provide trustworthiness to allow readers to make their own assessment of transferability. While every attempt was made to interview participants using video conferencing, due to internet bandwidth issues, some had to be interviewed using the telephone. This may have limited the non-verbal communication, impacting on the quality of the data collection. Despite this, careful listening was used as a mitigation strategy enabling the researcher to note rapid speech and changes in voice tone. Additionally, monetary incentives were used to facilitate participation in the semi-structured interviews which can be a limitation as it may minimise refusals to participate, however it must be noted that in this study a total of 84 individuals were contacted to participate with four declining to participate and 60 not responding.

## Conclusion

This study provides insight into the challenges of social isolation faced by many Australians during the COVID-19 pandemic. The results of this study have indicated that a lack of social capital prior to the pandemic has led to negative impacts including loneliness, and social isolation resulting in poor wellbeing during the pandemic. This has been exacerbated by existing and amplified social determinants of health such as loss of employment, income, gender, remoteness, and lack of social support. The findings highlight the need for interventions to increase social support, social cohesion, and social connectedness among Australians to enhance their overall wellbeing immediately and long term. Multiple and multilevel interventions aimed at a coordinated response to building networks that promote social participation and support among those with limited social capital are necessitated. This includes building social capital through involvement in community centres, exercise groups, partnerships with refugee leaders, neighbourhood programs and fostering intergenerational social capital programs. Social capital plays an enormous role in wellbeing and health, with this study identifying that the need for human connection is high therefore, interventions focussed on building social capital should be a priority. However, further research is required to develop optimal methods on implementing social capital interventions.

## Data Availability

The data that support the findings of this study are available on request from the corresponding author. The data are not publicly available due to privacy or ethical restrictions.
